# Voluntary Wheel Running in Old C57BL/6 Mice Reduces Age-Related Inflammation in the Colon but Not in the Brain

**DOI:** 10.3390/cells11030566

**Published:** 2022-02-06

**Authors:** Marie-Luise Ederer, Madlen Günther, Lena Best, Julia Lindner, Christoph Kaleta, Otto W. Witte, Rowena Simon, Christiane Frahm

**Affiliations:** 1 Hans Berger Department of Neurology, Jena University Hospital, Am Klinikum 1, 07747 Jena, Germany; Marie-Luise.Ederer@med.uni-jena.de (M.-L.E.); Madlen.Guenther@med.uni-jena.de (M.G.); julia.lindner@med.uni-jena.de (J.L.); otto.witte@med.uni-jena.de (O.W.W.); rowena.simon@med.uni-jena.de (R.S.); 2 Institute for Experimental Medicine, Kiel University, 24105 Kiel, Germany; l.best@iem.uni-kiel.de; 3 Department of Ophthalmology, Jena University Hospital, Am Klinikum 1, 07747 Jena, Germany; c.kaleta@iem.uni-kiel.de

**Keywords:** inflammaging, aging, social isolation, Barnes maze, hippocampus, intestine, gut-brain axis, spatial learning, physical activity

## Abstract

Inflammation is considered a possible cause of cognitive decline during aging. This study investigates the influence of physical activity and social isolation in old mice on their cognitive functions and inflammation. The Barnes maze task was performed to assess spatial learning and memory in 3, 9, 15, 24, and 28 months old male C57BL/6 mice as well as following voluntary wheel running (VWR) and social isolation (SI) in 20 months old mice. Inflammatory gene expression was analyzed in hippocampal and colonic samples by qPCR. Cognitive decline occurs in mice between 15 and 24 months of age. VWR improved cognitive functions while SI had negative effects. Expression of inflammatory markers changed during aging in the hippocampus (*Il1a*/*Il6*/*S100b*/*Iba1*/*Adgre1*/*Cd68*/*Itgam*) and colon (*Tnf*/*Il6*/*Il1ra*/*P2rx7*). VWR attenuates inflammaging specifically in the colon (*Ifng*/*Il10*/*Ccl2*/*S100b*/*Iba1*), while SI regulates intestinal *Il1b* and *Gfap*. Inflammatory markers in the hippocampus were not altered following VWR and SI. The main finding of our study is that both the hippocampus and colon exhibit an increase in inflammatory markers during aging, and that voluntary wheel running in old age exclusively attenuates intestinal inflammation. Based on the existence of the gut-brain axis, our results extend therapeutic approaches preserving cognitive functions in the elderly to the colon.

## 1. Introduction

The decline of cognitive functions and the prevalence of neurodegenerative disorders are among the greatest health threats of old age [1]. With the continuing rise in life expectancy and the accompanying increase in the population of the elderly, the situation is likely to worsen. The number of people affected by dementia will double every 20 years to 115.4 million worldwide by 2050 [2]. This demographic change demonstrates the vital importance of the maintenance of cognitive performance and prevention of dementia in old age for society.

The causes of age-related cognitive impairment are complex and difficult to fathom. Studies in this regard are increasingly focusing on the role of inflammaging, defined as age-related chronic and low-grade systemic inflammation. At the molecular level, immune- and inflammation-related genes are activated during aging and predispose the brain to neurodegenerative processes [3,4,5]. Increased expression of inflammation-related genes was also detected specifically in the hippocampus of aged mice [6]. The hippocampus (HC), which is involved in various cognitive tasks in both rodents and humans, is highly sensitive to numerous forms of stress and has been shown to be the most sensitive brain region in cognitive aging. Consistent with this, functional imaging techniques detect age-related changes in the structure, metabolism, and functional connectivity of the hippocampal formation that potentially affect cognitive abilities [7,8].

The causes of inflammaging, with their negative effects on the brain and cognitive abilities, are not yet conclusively understood. The gut-brain axis has become the focus of scientific attention in recent years, also with regard to influencing processes of aging. Moreover, gastrointestinal disorders are observed more frequently in the elderly [9], particularly as comorbidities to neurodegenerative diseases such as Alzheimer’s or Parkinson’s disease [10,11]. The enteric nervous system (ENS), a main regulatory system for gastrointestinal function, is composed of neurons and enteric glial cells. These neural-like intestinal cells show morphological and molecular similarities to brain cells, which is also reflected in overlapping cell marker expression [12]. For example, GFAP and S100B are used to identify subsets of enteric glia [13]. It is increasingly accepted that proper immune function of the gut is necessary for brain function and, conversely, that ENS dysfunction and inflammatory disease sensitize to neurodegeneration. This was impressively confirmed in a recent study, which identified intestinal risk genes for inflammatory and extra-intestinal diseases and suggests an involvement of these ENS genes in neurodegenerative processes [14]. Yet, there are still far too few studies focusing on the processes underlying inflammaging in the gut and how they can be modified by targeted interventions.

It is well accepted that physical activity improves overall immune function and reduces the incidence of inflammatory diseases [15]. Evidence from both animal and human studies underscores the role of physical activity in modifying metabolic, structural, and functional processes of the brain. Moreover, many studies show that an active lifestyle with adequate physical activity is a prerequisite for maintaining cognitive performance [16] and cerebral plasticity in the elderly [17].

In addition to a direct effect of physical activity on the brain, the existence of the gut-brain axis gives reason to assume that exercise also affects the multiple functions of the gut and its aging processes, which in turn may have a positive effect on cognitive functions. Compared to the brain, much less is known about the effects of physical activity on the gut and its immune system, although some positive effects on inflammatory bowel disease have already been described [18,19]. The influence of exercise on the integrity of the intestinal barrier is currently controversial and there is a need for more studies [20].

Social isolation (SI) in old age is also a risk factor for cognitive decline and elderly people are especially affected by SI more often [21]. That SI impairs cognition has already been described in studies in humans and animals [22,23]. However, there are few studies in model organisms of aging in which the effects and underlying molecular mechanisms of SI have been investigated. Given the need for SI in the current COVID-19 pandemic [24], the question of age-related effects of isolation on cognition and possible gut involvement becomes even more important.

Aging is a multi-tissue process, which suggests that brain aging and decline in cognition are driven by systemic processes. The existence of the gut-brain axis implies that various gut functions can be modulated and may contribute to altered cognitive function. Understanding such mechanisms holds the potential to target cognitive abilities through gut physiology. How physical activity and social isolation in old age affect cognitive function and inflammation in the brain and colon is poorly understood. As a baseline, male C57BL/6 mice at five different ages (3, 9, 15, 24, and 28 months) were analyzed. Cognitive function was assessed by a spatial learning and memory test using the Barnes maze task. At the molecular level, hippocampal tissue was analyzed for transcript expression of cytokines and chemokines (*Tnf*, *Ifng*, *Il1a*, *Il1b*, *Il6,* *Il10*, *Il1ra*, *Ccl2*, *Ccl3*), markers for activated astrocytes (*Gfap*, *S100b*), and microglia (*Iba1*, *Adgre1*, *Cd68*, *Itgam*, *P2rx7*). Colon tissue was examined for the same markers as the brain and for genes involved in gut barrier function (*Ocln, Cdh1, Tjp1, Cldn1, Muc2*) [25], which were also found altered in diseases associated with intestinal inflammation [26,27,28]. Changes in gene expression were compared across ages, after voluntary wheel running (VWR), and as a result of social isolation.

## 2. Materials and Methods

### 2.1. Animals

Male C57BL/6 mice were bred in the Central Experimental Animal Facility (ZET) at Jena University Hospital, Jena, Germany. Mice were maintained at 22 ± 2 °C on a 14 h/10 h day-night cycle and at a relative humidity of 55 ± 10%. Mice had unlimited access to water and food (ssniff mouse V1534-300, ssniff Spezialdiäten GmbH, Soest, Germany). Mice at different ages (3, 9, 15, 24, and 28 months), mice exposed to a voluntary running wheel (20 months old at testing), and corresponding control groups (20 months) including mice in social isolation were used (n = 138 in total). All studies were performed in strict compliance with the recommendations of the European Commission for the protection of animals used for scientific purposes and with the approval of the local government Thüringer Landesamt für Verbraucherschutz, Germany (license 02-024/15 and UKJ-19-014). Experiments are in accordance with the ARRIVE guidelines.

### 2.2. Voluntary Wheel Running and Social Isolation

At 18 months of age, mice were individualized and placed in transparent plastic cages with a running wheel (35.5 × 23.5 × 20 cm, Single Activity Wheel Chamber model 80820, Lafayette Instrument, Lafayette, IN, USA) for 8 weeks. The mice were allowed to use the wheel voluntarily. Single housing during running was necessary to record the individual performance per mouse using Scurry Activity Monitoring Software (Lafayette Instrument). Individualized mice were randomly assigned as isolated runners or isolated controls (hereinafter also referred to as the socially isolated group). Weight, health status, and spontaneous behavior were monitored weekly. After 6 weeks of VWR or SI, a 15 day behavior-test period followed. During this period, mice remained in their respective conditions. To control for the effect of isolated housing, a second control group with standard group housing was assigned. In summary, SI mice are compared with the VWR group (running effect) and with group housing mice (social isolation effect).

### 2.3. Barnes Maze Test

Behavioral tests were conducted to assess the effect of age (3, 9, 15, 24, and 28 months, n = 16–17 per age) and VWR (isolated runners, isolated and group-housed controls, 20 months at testing, n = 18–20 per condition) on cognitive abilities. Mice were familiarized with the experimental room and experimenter prior to the test to reduce general stress and anxiety. Our maze consisted of a circular platform 90 cm in diameter with 20 evenly spaced holes in the periphery, each 5 cm in diameter, and was placed 1 m high. The position of the escape box was randomly assigned to each mouse and kept constant during the experiment, except for the reversal test. Bright light served as an aversive stimulus. Illumination was set to 1200 lux at the center of the platform and at least 900 lux at the periphery. Distinct visual cues were placed on the walls to facilitate orientation in the room. At the beginning of each trial, the mouse was placed in a transparent cylinder at the center of the platform for 15 s before tracking began. During a single habituation trial, the mice were allowed to explore the Barnes maze platform for 120 s and to find the escape box. The training phase started three days later. For each training trial, mice were given 240 s to find the target hole. If not found within this time, mice were guided gently to their target hole. After voluntarily entering the escape box, the hole was covered and the mouse was left undisturbed for 30 s inside the box before being placed back into the home cage. The experimental setup was cleaned with 70% ethanol (*v*/*v*) (Nordbrand Nordhausen GmbH, Nordhausen/Harz, Germany) after each mouse. The training and test protocol used here was modified based on previous publications [29]: we performed three trials per day with an inter-trial interval of 60 min over 6 training days. For the probe trial, the escape box was removed and all holes were closed. The retention test took place 4 days after the probe trial, with the escape box set back in its original position. For the reversal test, the position of the escape box was changed by 180°. The probe trial, retention test, and reversal test were each performed as a single 240 s trial. All experiments were recorded and maximum velocity was automatically calculated by the video tracking system EthoVision^®^XT 6.1 software (Noldus, Wageningen, Netherlands). Primary latency (time to locate the target hole) was measured during the trial assessment. In addition, for the probe trial, the success rate in finding the target hole and the duration on the original target hole were analyzed, and for the reversal test, the success rate in finding the reversal hole was analyzed.

The spatial search strategies chosen by the mice in all trials and tests were analyzed using the Barnes maze unbiased strategy (BUNS) classification algorithm developed by Illouz et al. [30]. The EthoVision export files, which include the time-stamped x-y tracking coordinates per mice per trial, were imported into the BUNS application. To avoid classification artifacts due to the free-roaming of the mice on the platform after they found the target hole, tracking data were cleared after the primary latency timestamp prior to BUNS analysis for all runs except the probe trial. Each training trial and test was assigned to 1 of 6, and for the probe trial to 1 of 3 possible search strategies, which are rated with a numerical value defined as the cognitive score: highly spatial to non-spatial strategy: direct = 1, corrected = 0.75, long correction = 0.5, focused search = 0.5, serial = 0.25, and random = 0 [30]; probe trial highly spatial to non-spatial: focused search = 1, serial = 0.5, serial-random = 0.167. For mice who did not find the target hole during the probe trial, the cognitive score was set to 0. During the training period, a daily mean of the cognitive score was calculated for each animal.

Estimated training time (ETT) was calculated as a single measure of learning ability for each individual mouse. During the training period, primary latency follows a logarithmic decay. For each mouse, a natural logarithmic regression of the form −ln(y) = mx + t was fitted to the 18 primary latency measurements as a function of training day via the vglm function of the R-package VGAM (version 1.1-5). The use of the tobit model family accounted for the censored nature of primary latency by employing the parameters Upper = −ln(1) for 1 s as a minimum test time, Lower = −ln(240) for test abortion after 240 s, and model initialization via imethod = 2. ETT was then calculated as ETT = −t/m (m: slope, t: y-intercept), which corresponds to the training days required for a mouse to reach a perfect primary latency of 1 sec. Outliers (15 months: 2, 24 months: 2, 28 months: 1 animals) and negative ETT values from “non-learners” (2 months: 1, 9 months: 1, 15 months: 2, 24 months: 2, 28 months: 5 animals) were winsorized to the 95% quantile, corresponding to a value of 53.3 (function Winsorize with the parameter probs = c(0, 0.95) from the R-package DescTools version 0.99.43). Similarly, for mice where the calculation of the logarithmic regression failed due to too many aborted tests, the maximum ETT value of 53.3 was assumed (28 months: 1 animal).

### 2.4. qPCR Analysis

qPCR analysis was applied to test for the expression of preselected genes (supplemental information Tables S1 and S2). For this purpose, mice from the 3 months age group (HC: n = 9 (*Itgam* n = 8); colon: n = 10), the 20 months age group (aging effect, HC, and colon: n = 10 each), as well as 20 months old runners (HC: n = 10 (*Ccl3* n = 9); colon: n = 10) and their 20 months old isolated controls (HC: n = 9 (*Itgam* n = 8); colon: n = 10) were used. Mice were sacrificed five days after completion of the Barnes maze test by cervical dislocation to isolate the right HC and a part of the colon from the same mice. Total RNA was isolated using the phenol/chloroform extraction method [31]. RNA quantity and quality were determined spectrophotometrically using an ND-1000 (Nanodrop, Wilmington, DE, USA) and subsequently analyzed by a Qiaxcel Advanced System (Qiagen GmbH, Hilden, Germany). Total RNA was transcribed into cDNA (RevertAid First Strand cDNA Synthesis Kit, Thermo Fisher Scientific, Waltham, MA, USA). The qPCR was performed with Brilliant III SYBR^®^Green QPCR Master Mix (Agilent Technologies, Santa Clara, CA, USA) and specific mouse primers (biomers.net GmbH, Ulm, Germany, Table S1). Amplification was performed using Rotor-Gene 6000 cycler (Qiagen GmbH) applying the following cycle conditions: 3 min polymerase activation (95 °C) followed by 40 amplification cycles (95 °C for 10 s, 60 °C for 15 s). In a previous study, we confirmed *Gapdh* and *Hmbs* as suitable housekeeping genes in aged mice [32]. The mRNA transcript ratios were calculated using the Pfaffl equation [33].

### 2.5. Protein-Protein Association and Gene Ontology Analyses

Protein-protein association networks were constructed with stringDB version 11.5 (string-db.org accessed on 15 November 2021; [34]). Full string networks, where line thickness indicates interaction confidence, were created. Only input genes and “shell 2” interacting nodes (HC: 10, colon 7 interacting nodes) were visualized. Associations are shown only for an interaction score of at least 0.4. The interaction score was calculated based on experimental interactions, pathway databases, or co-expression evidence. Queried protein symbols were IL1A, IL6, S100B, IBA1 (AIF1), ADGRE1 (EMR1), CD68, ITGAM for HC and TNF, IL6, IL1RA (IL1RN), P2RX7, MUC2, IFNG, IL10, CCL2, S100B, IBA1 (AIF1), IL1B, GFAP for colon. Gene ontology tables were employed to determine functional associations of selected protein groups. Differential expressed genes were associated with biological functions via gene ontology analysis with the R-package clusterProfiler (version 4.0.5, function enricher). Mouse gene ontology database files were downloaded from purl.obolibrary.org/obo/go/go-basic.obo and current.geneontology.org/annotations/mgi.gaf.gz in 21st of September 2021. Query genes were stratified by tissue, experiment, and direction of expression regulation: hippocampal genes increased in aging (*Il1a, Il6, S100b, Iba1, Adgre1, Cd68, Itgam*), colonic genes increased in aging (*Tnf, Il6, Il1ra, P2rx7*) and colonic genes decreased following VWR (*Ifng, Il10, Ccl2, S100b, Iba1*). All genes tested via qPCR stratified by tissue were used as a background gene set.

### 2.6. Data Analysis

Statistical analyses were performed using IBM^®^SPSS^®^Statistics Version 26 (IBM Deutschland GmbH, Ehningen, Germany). The Barnes maze training period was analyzed using two-factor mixed-design ANOVA. Univariate ANOVA was used for the comparison of more than two groups for the probe trial, retention, and reversal test. Bonferroni correction was applied for multiple comparisons in ANOVA. Unpaired *t*-test was used for the comparison of two groups in the probe trial, retention test, and reversal test for numerical data. Nominal data were compared by Fisher’s exact test and, for more than two groups, by Pearson’s Chi-squared test with Fisher’s exact approach for post-hoc analysis [35]. Gene expression data were analyzed by the Mann-Whitney-U test. ETTs were compared between age groups by a Kruskal-Wallis test followed by Dunn’s test. Bonferroni correction was applied to correct for multiple comparisons (function dunnTest from R-package FSA version 0.9.1). Linear regression of ETTs to age was calculated with function lm from base R. Additionally, ETT was correlated to age with the function cor.test from base R and parameter method = “spearman”. All calculation steps were performed in R version 4.1.1, plotting was performed with package and function vioplot (version 0.3.7).

## 3. Results

### 3.1. Age-Related Cognitive Performance

Male C57BL/6 mice at different ages (3, 9, 15, 24, and 28 months) in group housing were subjected to a Barnes maze to assess their spatial learning and memory.

The primary latency significantly decreased during the training period at all ages (mixed ANOVA: effect of training day: 3 months: *F*_5, 385_ = 10.36, *p* < 0.001; 9 months: *F*_5, 385_ = 9.73, *p* < 0.001; 15 months: *F*_5, 385_ = 9.69, *p* < 0.001; 24 months: *F*_5, 385_ = 7.74, *p* < 0.001; 28 months: *F*_5, 385_ = 3.80, *p* = 0.002). While a significant training effect was already visible from day two for 3 (*p* = 0.003), 9 (*p* = 0.003), and 15 months old mice (*p* = 0.023), a significant improvement was demonstrated in the 24 (*p* = 0.003) and 28 months old mice (*p* = 0.025) from day four.

**Figure 1:**
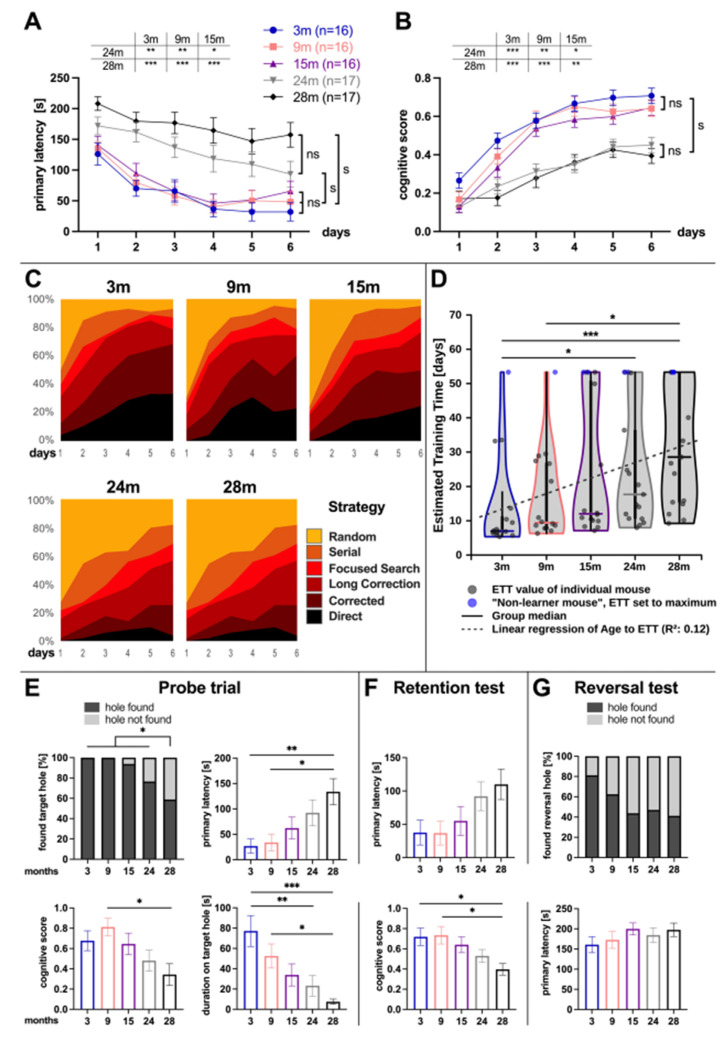
Effect of aging on cognitive function in male mice in the Barnes maze test. Aging significantly increased (**A**) primary latency and decreased (**B**) cognitive score during the training period. (**C**) Search strategies used during the training period differed between age groups. (**D**) The estimated training time (ETT) to achieve optimal test results differs between age groups and shows a significant association to age in linear regression. Significant effects of aging were also evident in the (**E**) probe trial and (**F**) retention test while the (**G**) reversal test showed no differences. ETT is shown as median, all other parameters are shown as mean ± SEM, the success rate in finding the target hole is presented as %. Specific significance levels for (**A**) primary latency and (**B**) cognitive scores are given in the tables above the graph. The training period was tested with a mixed ANOVA (A-B) and with a Kruskal-Wallis test (**D**). The probe trial, retention test, and reversal test were analyzed with a univariate ANOVA, and the success rate was tested with Fisher’s exact test, * *p* ≤ 0.05, ** *p* ≤ 0.01, *** *p* ≤ 0.001.

The comparison of the primary latency between different ages over the whole training period showed no difference between 3, 9, and 15 months old mice. Mice aged 24 and 28 months were not significantly different from each other, but significantly slower in finding the target hole than the other age groups (mixed ANOVA: age *F*_4, 77_ = 13.97, *p* < 0.001; multiple comparisons: 3 vs. 24: *p* = 0.002, 3 vs. 28: *p* < 0.001, 9 vs. 24: *p* = 0.009, 9 vs. 28: *p* < 0.001, 15 vs. 24: *p* = 0.037, 15 vs. 28: *p* < 0.001, 3 vs. 9: *p* = 1.00, 3 vs. 15: *p* = 1.00, 9 vs. 15: *p* = 1.00, 24 vs. 28: *p* = 0.29) (Figure 1A).

To provide a more fine-grained estimate of cognitive function that aggregates all test runs into a single value, we determined the estimated training time (ETT) from primary latency measures of the training phase for each mouse individually. The ETT indicates the training time (in days) each mouse would take to achieve optimal test results, that is, finding the hole within one second. Based on ETT, 3 months old animals were significantly better learners than 24 or 28 months old mice, and 9 months old were significantly better than 28 months old mice (3 vs. 24: *p* = 0.023, 3 vs. 28: *p* < 0.001, 9 vs. 28: *p* = 0.048). Animals that did not improve or worsened their primary latency times over the training period were termed “non-learners”. The count of those “non-learners” was higher in the older age groups (3 months: 1, 9 months: 1, 15 months: 2, 24 months: 2, 28 months: 5 animals). Overall, the ETT shows a significant association to the age of the tested mice in linear regression (*p* < 0.001, adjusted R² = 0.12) and Spearman’s correlation (*p* < 0.001, rho = 0.498) (Figure 1D).

To rule out a difference in the speed at which the mice moved in the Barnes maze, the maximum velocity of each age group was assessed on each of the six training days. Maximal velocity was significantly lower in the 28 months old mice compared to all other ages (mixed ANOVA: age *F*_4, 77_ = 6.42, *p* < 0.001; multiple comparisons: 3 vs. 28: *p* < 0.001, 9 vs. 28: *p* = 0.007, 15 vs. 28: *p* = 0.035, 24 vs. 28: *p* = 0.020, 3 vs. 9: *p* = 1.00, 3 vs. 15: *p* = 0.73, 3 vs. 24: *p* = 0.93, 9 vs. 15: *p* = 1.00, 9 vs. 24: *p* = 1.00, 15 vs. 24: *p* = 1.00). All other ages showed no significant differences from each other.

The spatial performance during training was further evaluated using the BUNS algorithm (Figure 1C). Young animals used more spatial search strategies (direct, corrected), whereas during aging the rate of random searches increased. For quantitative analysis, the search strategies were rated with a cognitive score and compared between the groups (Figure 1B). This velocity-independent parameter confirmed that 3, 9, and 15 months old mice did not differ from each other in cognitive performance but were significantly better than 24 and 28 months old mice, which did not differ from each other (mixed ANOVA: age *F*_4, 77_ = 11.07, *p* < 0.001; multiple comparisons: 3 vs. 24: *p* < 0.001, 3 vs. 28: *p* < 0.001, 9 vs. 24: *p* = 0.004, 9 vs. 28: *p* = 0.001, 15 vs. 24: *p* = 0.038, 15 vs. 28: *p* = 0.007, 3 vs. 9: *p* = 1.00, 3 vs. 15: *p* = 0.74, 9 vs. 15: *p* = 1.00, 24 vs. 28: *p* = 1.00). 

At day seven, the first day after training, the probe trial with all holes closed was performed to test short-term memory. We found an age-dependent success rate in finding the original hole position with a significant lower success rate for 28 months old mice (3 months: 100%, 9 months: 100%, 15 months: 93.8%, 24 months: 76.5%, 28 months: 58.8%, Pearson’s Chi-squared test: *p* < 0.002; Fisher’s exact approach for post-hoc analysis: 28 vs. 3, 9, 15, and 24 months: *p* = 0.002) (Figure 1E). The 3 and 9 months old mice were significantly faster in reaching the original target hole position (primary latency) than the 28 months old mice (univariate ANOVA: age *F*_4, 77_ = 4.29, *p* = 0.003; multiple comparisons: 3 vs. 28: *p* = 0.007, 9 vs. 28: *p* = 0.014). This parameter alone does not provide reliable information about cognitive performance, as the maximum velocity also differed significantly between the same groups (univariate ANOVA: age *F*_4, 77_ = 4.95, *p* = 0.001; multiple comparisons: 3 vs. 28: *p* = 0.002, 9 vs. 28: *p* = 0.013). The velocity-independent cognitive score (3 months: 0.68 ± 0.10, 9 months: 0.81 ± 0.09, 15 months: 0.65 ± 0.11, 24 months: 0.48 ± 0.10, 28 months: 0.34 ± 0.11) confirms that 9 months old mice showed a superior spatial performance compared to 28 months old mice (univariate ANOVA: age *F*_4, 77_ = 3.29, *p* = 0.015; multiple comparison 9 vs. 28: *p* = 0.015). We further compared the time mice spent at the original target hole position during the 240 s time interval and found that young mice remained at this location significantly longer (univariate ANOVA: age *F*_4, 77_ = 6.19, *p* < 0.001; multiple comparisons: 3 vs. 24: *p* = 0.007, 3 vs. 28: *p* < 0.001, 9 vs. 28: *p* = 0.043). The retention test on day 11 with the escape box open at the original position assessed long-term memory (Figure 1F). The cognitive score was significantly higher in the 3 and 9 months old compared to the 28 months old group (univariate ANOVA: age *F*_4, 77_ = 3.63, *p* = 0.009; multiple comparisons: 3 vs. 28: *p* = 0.031, 9 vs. 28 *p* = 0.020). Although a variance in the univariate ANOVA (age *F*_4, 77_ = 2.59, *p* = 0.043) was significant for primary latency, in the multiple comparisons no group differences were shown after Bonferroni correction (Figure 1F). Maximum velocity did not differ between the groups (univariate ANOVA: age *F*_4, 75_ = 1.42, *p* = 0.24).

The reversal test performed on day twelve, with the escape box opened 180° from the original position, assessed cognitive flexibility. The rate of finding the new hole position was highest in the 3 months old group (3 months: 81.3%, 9 months: 62.5%, 15 months: 43.8%, 24 months: 47.1%, 28 months: 41.2%), but the Pearson’s Chi-squared test did not show differences between the age groups (*p* = 0.12) (Figure 1G). The latency to reach the reversal position did not differ between the groups (univariate ANOVA: age *F*_4, 77_ = 0.82, *p* = 0.51).

For all parameters tested here, no significant impairment of spatial learning and memory was detected in male mice up to 15 months of age (Figure 1). In 24 months old animals, all tested parameters of learning (training phase) and some short-term memory parameters (probe trial) were impaired compared with younger age groups. These differences were even more pronounced in the 28 months old mice (Figure 1A–C). Interestingly, we found no further significant loss of cognitive function between 24 and 28 months old mice (Figure 1). Cognitive flexibility, as assessed with the reversal test, showed no age dependence with our Barnes maze protocol.

### 3.2. Voluntary Wheel Running Improves Cognitive Function in Aged Isolated Mice

**Figure 2:**
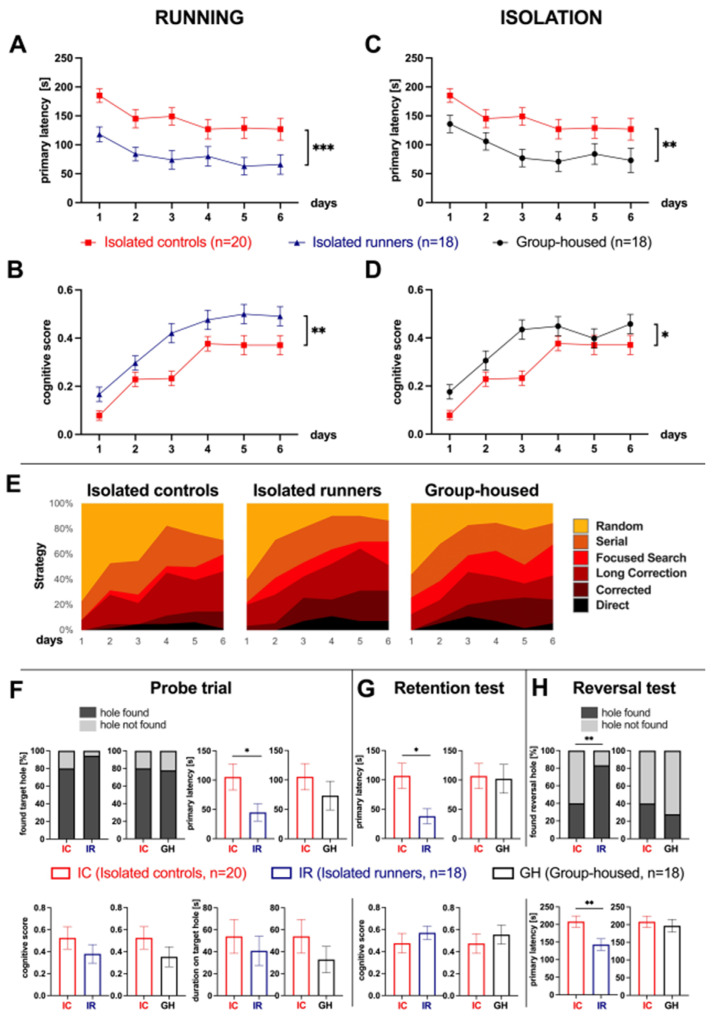
Effect of running and isolation on cognitive functions in male mice in the Barnes maze test. Isolated runners and group-housed mice were each compared to the same group of isolated controls. Running decreased (**A**) primary latency and increased (**B**) the cognitive score during the training period. (**C**) Isolation increased primary latency and decreased (**D**) the cognitive score. (**E**) Search strategies differed between isolated runners, isolated controls, and group-housed mice. (**F**) The probe trial, (**G**) the retention test, and (**H**) the reversal test were significantly different with respect to the primary latency after VWR. Cognitive score and primary latency are shown as mean per day ± SEM, mixed ANOVA. All other parameters are shown as mean ± SEM, *t*-test. The success rate in finding the target hole is presented as % and was tested with Fisher’s exact test. * *p* ≤ 0.05, ** *p* ≤ 0.01, *** *p* ≤ 0.001.

Mice at 18 months of age were tested for their cognition after 2 months of VWR at 20 months of age. The mice ran an average of 1.81 ± 0.32 km/day. As the mice had to be isolated (isolated runners: IR) to test their individual running performance, mice in the age-matched control group were also isolated (isolated controls: IC). Both mice exposed to VWR (IR) and socially isolated controls (IC) showed an improvement in the primary latency over the training period (mixed ANOVA: effect of training day: isolated runners *F*_5, 180_ = 3.52, *p* = 0.005; isolated controls *F*_5, 180_ = 5.00, *p* < 0.001). The running mice reached the target hole significantly faster than the isolated controls (mixed ANOVA: housing: *F*_1, 36_ = 12.00, *p* = 0.001) (Figure 2A).

No significant differences between the groups were found for the maximum velocity during the whole training period (isolated runners: 69.7 ± 3.4 cm/s (mean ± SEM), isolated controls: 63.7 ± 3.3 cm/s; mixed ANOVA: housing *F*_1, 36_ = 1.65, *p* = 0.21).

The better spatial performance during training of the running compared to isolated mice was confirmed by a significantly higher cognitive score, with both groups, IC and IR, improving over time (mixed ANOVA: housing: *F*_1, 36_ = 10.47, *p* = 0.003; training day: *F*_5, 185_ = 26.61, *p* < 0.001) (Figure 2B,E).

In the probe trial, mice from the running group reached the original target hole position in a shorter time interval (primary latency: isolated runners: 44.8 ± 14.8 s, isolated controls: 105.4 ± 22.3 s, unpaired *t*-test: *t*_32_ = 2.26, *p* = 0.031) (Figure 2F). As there were also differences in the maximum velocity (isolated runners: 91.3 ± 5.6 cm/s, isolated controls: 72.1 ± 7.3 cm/s, unpaired *t*-test: *t*_34_ = 2.09, *p* = 0.044), we tested the cognitive score (isolated runners: 0.38 ± 0.09, isolated controls: 0.53 ± 0.10, unpaired *t*-test: *t*_35_ = −1.09, *p* = 0.28) and found no group differences. The success in finding the original hole position (isolated runners: 94.4%, isolated controls: 80%, Fisher’s exact test: *p* = 0.34) and the duration at this position (isolated runners: 40.8 ± 13.3 s, isolated controls: 54.0 ± 15.3 s, unpaired *t*-test: *t*_36_ = 0.64, *p* = 0.53) did not differ between the groups.

In the retention test, the mice of the running group found the original target hole faster (primary latency: isolated runners: 38.0 ± 12.9 s, isolated controls: 106.8 ± 21.5 s, unpaired *t*-test: *t*_31_ = 2.75, *p* = 0.010) (Figure 2G). No differences in the maximum velocity were found (isolated runners: 63.0 ± 7.4 cm/s, isolated controls: 52.8 ± 6.1 cm/s, unpaired *t*-test: *t*_36_ = 1.07, *p* = 0.29). The cognitive score did not differ between the groups (isolated runners: 0.57 ± 0.06, isolated controls: 0.48 ± 0.09, unpaired *t*-test: *t*_33_ = 0.88, *p* = 0.38).

In the reversal test the primary latency to find the reversal escape box position was significantly shorter in the running group (isolated runners: 142.8 ± 16.5 s, isolated controls: 208.3 ± 16.1 s, unpaired *t*-test: *t*_36_ = 2.84, *p* = 0.007) (Figure 2H). The maximum velocity was also higher (isolated runners: 93.9 ± 4.5 cm/s, isolated controls: 70.2 ± 8.2 cm/s, unpaired *t*-test: *t*_29_ = 2.55, *p* = 0.016). Therefore, it is important to consider another parameter to prove that the effect of the latency is not only due to a higher speed of the running group. We found a group difference in the success rate in finding the new escape box position (isolated runners: 83.3%, isolated controls: 40%, Fisher’s exact test: *p* = 0.009) (Figure 2H) indicating higher cognitive flexibility following running. 

### 3.3. Social Isolation Decreases Cognitive Function in Aged Mice

To test the effect of isolating the mice on their cognitive performance, isolated mice were compared with age-matched 20 months old mice in standard group housing. Group-housed mice showed a significantly shorter primary latency (mixed ANOVA: housing *F*_1,36_ = 7.51, *p* = 0.009) (Figure 2C) and a higher cognitive score during the training period (mixed ANOVA: housing *F*_1, 36_ = 7.28, *p* = 0.011) (Figure 2D, E), while the maximum velocity did not differ (isolated controls: 63.7 ± 3.3 cm/s, group-housed: 70.7 ± 3.4 cm/s; mixed ANOVA: housing *F*_1, 36_ = 2.18, *p* = 0.15).

The parameter tested for the probe trial showed no group differences (primary latency: isolated controls: 105.4 ± 22.3 s, group-housed: 73.3 ± 24.3 s, unpaired *t*-test *t*_36_ = 0.97, *p* = 0.34; duration on original hole: isolated controls: 54.0 ± 15.3 s, group-housed: 32.8 ± 12.0 s, unpaired *t*-test: *t*_36_ = 1.07, *p* = 0.29; cognitive score: isolated controls: 0.53 ± 0.10, group-housed: 0.35 ± 0.09, unpaired *t*-test: *t*_36_ = 1.27, *p* = 0.21; success in finding the target hole: isolated controls: 80%, group-housed: 77.8%, Fisher’s exact test: *p* = 1.00) (Figure 2F). The same applies to the retention test (primary latency: isolated controls: 106.8 ± 21.5 s, group-housed: 102.1 ± 24.5 s, unpaired *t*-test: *t*_36_ = 0.15, *p* = 0.89; cognitive score: isolated controls: 0.48 ± 0.09, group-housed: 0.56 ± 0.09, unpaired *t*-test: *t*_36_ = 0.65, *p* = 0.52) and the reversal test (primary latency: isolated controls: 208.3 ± 16.1 s, group-housed: 196.8 ± 17.9 s, unpaired *t*-test: *t*_36_ = 0.48, *p* = 0.64, success rate in finding the new hole position: isolated controls: 40%, group-housed: 27.8% Fisher’s exact test: *p* = 0.51) (Figure 2G,H). These results show that SI in old mice leads to impaired cognitive abilities especially during the training period, while short-term and long-term memory as well as cognitive flexibility were not found to be affected in our experimental setup.

When comparing all 20 months old groups of mice with each other, running mice and mice housed in groups do not differ significantly from each other, while the group of isolated mice performed significantly worse in the tests (mixed ANOVA of primary latency: housing *F*_2, 53_ = 6.94, *p* = 0.002; multiple comparisons: isolated runners vs. group-housed: *p* = 1.00, isolated runners vs. isolated controls: *p* = 0.003, group-housed vs. isolated controls: *p* = 0.017).

### 3.4. Effect of Aging on Gene Expression in the Hippocampus and Colon

**Figure 3:**
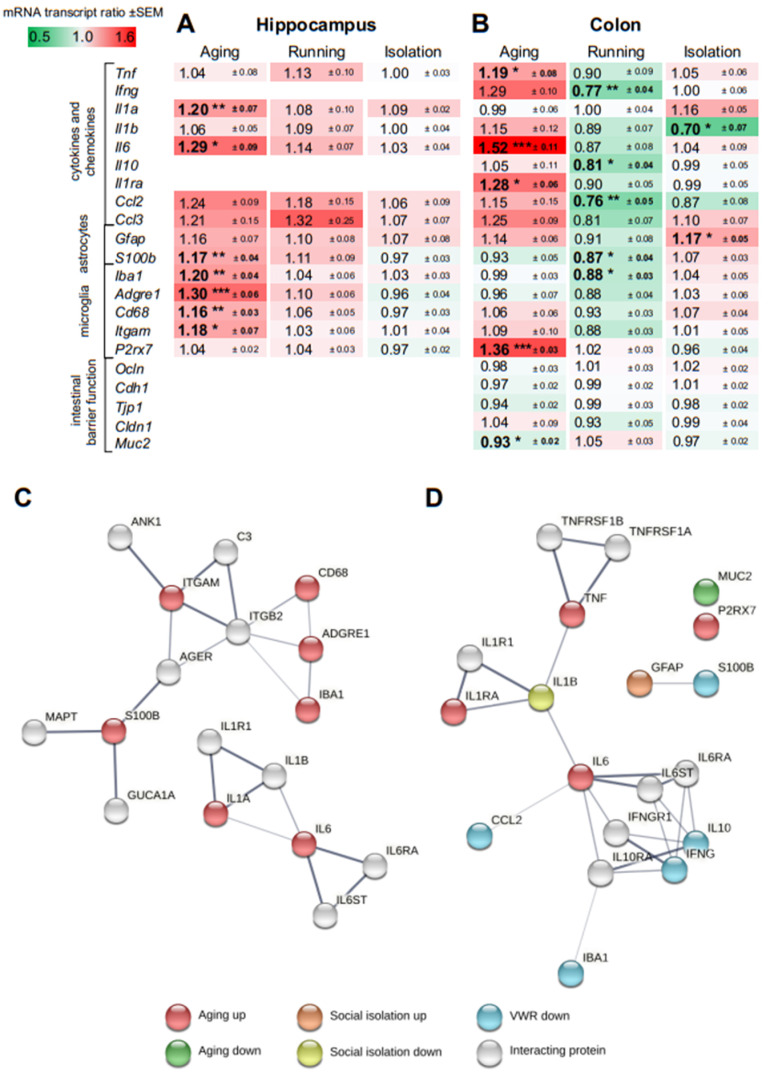
Transcript expression in the (**A**) HC and (**B**) colon after aging, running, and social isolation. For aging, 20 versus 3 months old animals were compared. For running, 20 months old isolated runners were compared with isolated control mice; for isolation, 20 months old isolated mice were compared with group-housed mice. The mRNA transcription ratios are displayed as geometric mean ± SEM. Statistically significant ratios are in bold, Mann-Whitney-U test, * *p* ≤ 0.05, ** *p* ≤ 0.01, *** *p* ≤ 0.001, n = 8–10. Specific *p*-values for each significant mRNA transcription ratio are given in the text. Protein-protein association network (STRING v11) of significantly differentially expressed genes in the (**C**) HC and (**D**) colon. The network is based on experimental interaction, pathway databases, or co-expression evidence. Confidence in associations is displayed by line thickness.

To assess the grade of inflammaging in the 20 months old mice group, they were compared with 3 months old mice, both group-housed. Hippocampal tissue was analyzed for transcript expression of pro-inflammatory cytokines (*Tnf*, *Ifng, Il1a*, *Il1b*, *Il6*), anti-inflammatory cytokines (*Il10*, *Il1ra*), and chemokines (*Ccl2*, *Ccl3*). We also examined markers for activated astrocytes (*Gfap*, *S100b*) and microglia (*Iba1*, *Adgre1*, *Cd68*, *Itgam, P2rx7*). Colon tissue was examined for the same markers as the brain and for genes involved in gut barrier function (*Ocln, Cdh1, Tjp1, Cldn1, Muc2*). *Ifng*, *Il10*, and *Il1ra* were insufficiently expressed in hippocampal tissue and therefore not considered in the analysis of hippocampal gene expression. The mRNA transcription ratios are displayed in Figure 3 as geometric mean ± SEM (20 vs. 3 months old mice).

In the HC, *Il1a* (increased by 20.19 ± 6.90%, *p* = 0.008), *Il6* (increased by 29.01 ± 9.17%, *p* = 0.013), *S100b* (increased by 17.06 ± 3.86%, *p* = 0.004), *Iba1* (increased by 19.87 ± 4.05%, *p* = 0.002), *Adgre1* (increased by 29.83 ± 5.54%, *p* < 0.001), *Cd68* (increased by 15.94 ± 2.83%, *p* = 0.004), and *Itgam* (increased by 18.22 ± 7.39%, *p* = 0.043) showed a significantly higher expression in 20 months old mice compared to 3 months old mice (Figure 3A). In the aging colon, *Tnf* (increased by 18.86 ± 7.69%, *p* = 0.023), *Il6* (increased by 51.68 ± 11.13%, *p* < 0.001), *Il1ra* (increased by 28.33 ± 6.37%, *p* = 0.011), and *P2rx7* (increased by 36.27 ± 3.29%, *p* < 0.001) were found up-regulated. *Muc2* was expressed less in the colon of 20 months old mice compared to 3 months old mice (decreased by 7.00 ± 1.53%, *p* = 0.029) (Figure 3B). All other genes that play a role in gut barrier function (*Ocln, Cdh1, Tjp1, Cldn1*) remained unchanged.

### 3.5. Effect of Voluntary Wheel Running on Gene Expression in the Hippocampus and Colon

To assess the impact of running on the expression of genes initially tested for their age effect, 20 months old isolated running mice were compared with 20 months old isolated control mice (mRNA transcription ratios are displayed in Figure 3 as geometric mean ± SEM). No significant changes in gene expression were found in hippocampal tissue after VWR for 2 months (Figure 3A). In the colon, *Ifng* (decreased by 23.09 ± 4.28%, *p* = 0.004), *ll10* (decreased by 19.30 ± 3.62%, *p* = 0.011), *Ccl2* (decreased by 24.12 ± 4.91%, *p* = 0.009), *S100b* (decreased by 12.51 ± 4.09%, *p* = 0.023), and *Iba1* (decreased by 12.43 ± 3.39%, *p* = 0.043) were downregulated (Figure 3B).

### 3.6. Effect of Social Isolation on Gene Expression in the Hippocampus and Colon

To assess the effect of SI on the expression of the genes tested for their age effect, 20 months old isolated mice were compared with 20 months old group-housed mice (mRNA transcription ratios are displayed in Figure 3 as geometric mean ± SEM). For hippocampal tissue, no significant gene expression changes of the markers tested were found (Figure 3A). In the colon, *Il1b* was downregulated (decreased by 30.07 ± 6.92%, *p* = 0.023). *Gfap* was upregulated by social isolation (increased by 17.34 ± 5.27%, *p* = 0.029) (Figure 3B).

### 3.7. Protein-Protein Association and Gene Ontology Analyses

In the HC, significantly differentially expressed genes were exclusively found up-regulated during aging, and their protein products were grouped with interacting proteins into two clusters in a protein-protein association (PPA) analysis (Figure 3C). The bigger cluster centered around ITGAM is associated with biological processes relevant for amyloid-beta clearance (ITGAM, C3, ITGB2), learning or memory (AGER, S100B, MAPT), positive regulation of neuron death (ITGAM, AGER, MAPT), or microglial cell activation (ITGAM, IBA1, AGER). The smaller protein cluster centered around IL6 consists of proteins that are well known for their function in cytokine-mediated signaling pathways. No significant enrichment was found for these up-regulated genes in a gene ontology analysis.

Most genes with significant expression changes in the colon (aging, running, isolation) and interacting proteins were grouped into one main cluster by PPA (Figure 3D). The majority of these proteins are relevant in cytokine-mediated signaling (IL6, IL1R1, CCL2, IL1B, IL6RA, IL6ST, IFNGR1, IL10RA, TNFRSF1A). The main cluster shows two sub-clusters connected via the hub proteins IL1B and IL6 (Figure 3D). The upper sub-cluster is comprised of proteins that are all relevant in inflammatory responses, while some of them are additionally involved in the regulation of neuron apoptotic processes or the establishment of endothelial barriers (TNF, TNFRSF1A, IL1B). The bottom sub-cluster proteins are mainly involved cytokine-mediated signaling pathways (IL6, CCL2, IL6RA, IL6ST, IFNGR1, IL10RA) and in the positive regulation of N-Methyl-D-aspartate glutamate (NMDA) receptor activity (IFNG, CCL2, IFNGR1).

Via gene ontology, genes that were up-regulated in the aging colon (Figure 3D, red) were associated with inflammatory responses (*Tnf, Il6, Il1ra, P2rx7*), whereas those genes which were down-regulated in VWR mice (Figure 3D, blue) are involved in biological processes of positive regulation of NMDA glutamate receptor activity (*Ifng, Ccl2*) or negative regulation of neuron apoptotic processes (*Il10, Ccl2*) (Table S3).

## 4. Discussion

### 4.1. Age-Related Cognitive Decline

This study was initiated to find out when impairments in cognitive traits occur in aging male C57BL/6 mice in order to provide VWR during this period. The aim is to maintain cognition in old age through a physical intervention started late in life and to identify underlying systemic mechanisms, particularly with regard to the gut-brain axis.

We choose the Barnes maze test for analyzing male C57BL/6 mice at five different ages taken from the breeding facility at the University Hospital Jena for hippocampus-dependent spatial functions because it is less stressful than the Morris water maze test [36]. It should be emphasized that in contrast to many other studies, we tested various ages, including mice at a very old age. An age of more than 30 months is reached by only about 4% of mice [37].

The younger age groups (3, 9, and 15 months) did not reveal significant differences in all Barnes maze parameters tested (Figure 1). At 24 months, several tested parameters were impaired: the primary latency to find the escape box and the cognitive score during the training phase, and a shorter duration at the target hole in the probe trial (Figure 1). The 28 months old mice differed from the younger age groups (3, 9, and 15 months) in all tested parameters of the training phase (Figure 1A–D). In the probe trial, significant differences in the tested parameters were detected in 28 months old compared to 3 and 9 months old mice (Figure 1E). The same applies to the cognitive score in the retention test (Figure 1F). In contrast, the reversal test did not detect any significant differences between the age groups (Figure 1G). Remarkably, we found no further significant loss of cognitive function between 24 and 28 months old mice and also with regard to estimated training time (Figure 1). This is a significant finding, as it could also have been assumed that cognition continues to decline in very old animals. The test parameters during the training period (learning) and the probe trial (short-term memory) revealed the most significant cognitive changes depending on age. Thus, we were able to identify a time window between 15 and 24 months in which cognitive decline occurs in male C57BL/6 mice.

Our results are in agreement with other studies that have investigated spatial memory in male mice of different ages. For example, deficits in the Morris water maze task were found in 18–19 months old compared to 3 months old C57BL/6J-Nia mice [38]. Another group has found learning and memory deficits in C57BL/6 mice in different maze tasks when comparing 15 to 2 months old mice. Using the Y-maze alternation task, they revealed that aging causes a decline in short-term memory [39]. Using the Morris water maze task, the 15 months old mice exhibited significantly longer escape latency during training. In the spatial exploration test, which is comparable to our probe trial, the number of platform crossings and the time in the target quadrant were significantly lower in the old than in young, 2 months old mice, which were also characterized by a superior search strategy [39]. However, there are also deviations. For example, Bach and colleagues examined 3, 6, 12, and 18 months old C57BL/B6 mice with the Barnes maze test and found cognitive deficits as early as 12 months of age [40]. The age at which cognitive deficits appear depends on the strain and differs to some extent even within a strain [41], possibly due to differences in breeding conditions, and depending on the specific cognitive tests that are applied.

### 4.2. Effect of Running and Social Isolation on Cognitive Function

To use this time window when age-related impairments occur for interventions with the aim of halting cognitive decline, 18 months old mice were exposed to VWR for 8 weeks. Tests were performed in the 7th and 8th weeks. Running decreased the primary latency to the target hole and increased the cognitive score during the training period (Figure 2A,B). Moreover, the probe trial, the retention and the reversal test were significantly improved with respect to the primary latency after VWR (Figure 2F–H). These results generally confirm the assumption that old brains still respond to exercise, for review see [42]. Voluntary running has already been shown to have a positive effect on spatial learning in aged mice. After 5 weeks of running, 19 months old mice were tested in the Morris water maze and showed a faster acquisition (latency to platform) during training [43]. However, the positive effect also seems to depend on how long the running wheel is provided to the mice, as a 3 weeks running period in ~17 months old mice showed no benefit on reference or working memory when performing the radial-arm water maze task [44].

To control for the effect of 8 weeks of social isolation, we compared socially isolated mice to group-housed male C57BL/6 mice at the age of 20 months. SI leads to spatial learning impairments characterized by a longer primary latency to reach the target hole and a lower cognitive score assessed in the training period of the Barnes maze task, while short- and long-term memory (probe trial and retention test), as well as cognitive flexibility (reversal test), were not affected in our experimental test design. 

In a comparable experiment to ours, 20 months old male mice were tested with the Morris water maze after 8 weeks of isolation. In these mice, the escape latency to reach the platform was longer during the 4 day training phase. During the probe trial, both, the percentage of time spent in the target quadrant and the number of times crossing the platform area decreased. Thus, our findings are in agreement with these results. Search strategy, long-term memory, and cognitive flexibility were not examined [45].

In summary, we observed a cognitive decline in old mice between 15 and 24 months of age. VWR significantly improved cognitive performance in 20 months old mice, whereas SI resulted in a decline. The magnitude of improvement after VWR and the magnitude of the decline in cognition after SI are approximately the same, but this does not mean that running compensates for the negative deficits of SI. This would require starting a new experimental approach, first SI and then testing whether subsequent VWR leads to a reversal of the negative effects.

### 4.3. Gene Expression Changes during Aging, Following Running and Social Isolation in the Hippocampus and Colon

Assessing the degree of inflammation in 20 months old mice, we found significantly higher expression *of Il1a*, *Il6*, *S100b*, *Iba1*, *Adgre1*, *Cd68,* and *Itgam* transcripts in the HC and of *Tnf*, *Il6*, *Il1ra*, and *P2rx7* transcripts in the colon tissue compared to 3 months old mice (Figure 3A,B). An increase in the transcript expression of cytokines, astroglial, and microglial activity markers in the brain, specifically in the HC, has already been published by us [6]. Which specific genes are increased and to what extent depends in particular on age and on the brain region being compared. In the colon, there is much less research on inflammaging. In a comparison of 16 ± 2 months and 2 months old male mice, *Tnf* and *Il6* were found to increase in the colon [46]. *P2rx7* was described to be upregulated in intestinal stem cells in 27 months old mice compared to 7–8 months old male mice [47]. The downregulation of *Muc2* we found is consistent with reduced mucus thickness in this age range [48]. In conclusion, we confirmed signs of inflammaging in the HC and were able to extend this phenomenon to the colon, where there has been little knowledge of inflammaging so far.

VWR did not significantly alter gene expression in the HC (Figure 3A). Agreeing with this, 10 weeks of VWR had no effect on the neuroinflammatory response to lipopolysaccharide (LPS) with respect to mRNA expression of *Tnf*, *Il1b*, and *Il6* in whole brains of 21 months old mice [49]. On the contrary, microglia isolated from the hippocampi of 24 months old rats after 6 weeks of VWR showed a reduced mRNA expression of *Tnf* and *Il1b* compared to controls, with and without LPS stimulation [50]. By analyzing colonic tissue, we revealed a significant decrease in *Ifng*, *ll10*, *Ccl2*, *S100b,* and *Iba1* (Figure 3B). Down-regulation of *Ifng* was also detected in colonic tissue of 2–3 months old mice after 6 weeks of VWR [51]. These mice were also characterized by a decrease in *Tnf*, which was not significantly changed in our aged mice, and an increase in the protective *Il10* [51], which we found downregulated in the aged colon after running (Figure 3B). Mice with induced colitis were subjected to forced moderate exercise, which led to an increase in cytokines such as *Tnf*, *Il1b*, and *Il10* in the colon and exacerbated colitis symptoms, whereas 30 days of VWR attenuated colitis in these mice and was associated with a decrease in *Tnf* [52]. We found *S100b*, a marker of reactive astrocytes in the brain that is also increased in intestinal inflammation [53], was decreased in the aged colon after running (Figure 3B). In a mouse model of accelerated aging, *Ccl2* was decreased in the small intestine of 11 to 12 months old male mice after 12 weeks of running [54]. In summary, VWR had no effect on inflammatory markers examined in the HC. Another study shows cell type-specific effects of regulation of inflammatory genes after VWR [50], implying that analysis of microglial cells, in particular, could be useful in this context. A different regime of VWR could also have an impact on the HC. Remarkably, we demonstrated that VWR is able to lower specific inflammatory markers in the aged colon, with no effects detected on markers of intestinal barrier integrity.

To understand the negative effects of SI on learning and memory, both HC and colon tissue of 20 months old mice were examined for inflammatory markers. We found none of the analyzed inflammatory genes altered in the aged HC following SI (Figure 3A). As has been shown before, *Il6*, *Il1b*, *Tnf*, *Itgam*, and *Bdnf* mRNA expression were not changed in the HC after one month of isolation in female mice beginning at 18 months [55] but protein expression of IL6, IL1B, and TNF was found upregulated and BDNF downregulated in HC of male mice at the same age but after a longer SI of 2 months [45]. We found *Gfap* significantly increased in the colon and *Il1b* unexpectedly decreased after SI (Figure 3B). In a model of chronic restraint stress in young 2 months old mice, GFAP^+^ enteric glia reactivity was higher in the myenteric plexus and lower in the lamina propria while IL1B protein expression was found increased [56]. In the colon of young 2 months old mice exposed to social defeat stress, various cytokines were downregulated (*Il1b*, *Il6*, *Il10*, *Tnf*, *Ifng*) which was explained by stress-induced immune suppression [57]. Protein expression of pro-inflammatory cytokines was found to be increased (TNF, IFNG) as well as decreased (IL10), and tight junction proteins (OCLN and TJP-1/ZO-1) in the colon of ~4 months old Wistar rats following a chronic restraint stress protocol, which was much more rigorous compared to our SI [58].

Protein-protein association networks were constructed for the significantly regulated genes in the hippocampus and colon using StringDB. The PPA analysis grouped proteins into two clusters. Similar to observations in the colon, one of the clusters can be attributed to inflammatory responses. This reflects the previously reported pro-inflammatory state of aging (inflammaging) which we previously found as part of the core signature of aging [59]. PPA analysis identified AGER and ITGB2 as important interacting proteins involved in cerebral processes such as learning or memory and amyloid-beta clearance, and are therefore of relevance for future analyses.

Although none of the differential expressed genes in the colon were found across the experimental conditions (aging, running, isolation), they are linked functionally via PPA or gene ontology. For instance, genes changed by running and another gene set changed in aging were both associated with the neuron apoptotic process. All up-regulated genes in aging can be attributed to the inflammatory response and thus, are part of inflammaging [59]. PPA analysis identified IL1B and IL6 as relevant hub-proteins. *Il1b*, which was down-regulated in socially isolated mice, is involved in endothelial barrier function and neuronal apoptosis together with TNF and TNFRSF1A. *Il6* expression was found to be increased in older mice and IL6 was identified as a hub between proteins whose mRNA was found down-regulated in VWR and social isolation, and which are associated with cytokine-mediated signaling. Although P2RX7 is not connected to other proteins in the PPA network, it is related to proteins of the main cluster functionally-wise as a master regulator of inflammation [60].

Taken together, changes in gene expression depend mainly on the type of social isolation stress and the age of the animals. The response of old mice to SI was less pronounced than expected from studies of young mice. This is consistent with a report that 24 months old mice did not respond significantly to SI and that SI had additive and synergistic effects with age [61]. Thus, the non-adaptive responsiveness of old, socially isolated mice could also accelerate aging and thereby increase its deleterious effects [61].

Our studies have shown that the old brain still responds malleably to interventions and demonstrates positive or negative effects on cognition depending on the type of intervention. The cognitive alterations following VWR and SI, that were measurable in the behavioral test, could not be directly linked to changes in inflammation-related genes in the HC, but remarkably, we found a positive effect on inflammaging in the colon after VWR. The next step is to clarify the relevance of these gene changes in the colon for cognitive functions.

What does it mean that VWR and SI have a significant impact on cognition, but no effect on the cerebral inflammation? Obviously, the main players of inflammaging in the aged hippocampus are not affected by our interventions and therefore cannot be considered as a direct cause for the revealed altered cognition. Interestingly, and this is our most important finding, we found a reduction of markers involved in inflammaging in the gut as a consequence of VWR. To what extent and whether the reduction of markers involved in inflammation in the gut contributes to better cognition cannot be answered by our study. Genome-wide screening in the hippocampus is planned to identify expression changes of cerebral cognition-related genes following VWR. The same analysis is planned for colon tissue, including 16S RNAseq and metabolome screening of the gut microbiome. It is known that the gut microbiome undergoes profound changes with aging [62] and plays an important role in aging processes and neurodegenerative diseases [63]. Furthermore, the gut microbiome is closely linked to cells of the colon. Certain bacterial genera correlate closely with the regulation of colonic gene expression [64]. Whether the expression changes in the colon are induced by a VWR-altered microbiome and whether specific microbiome-associated metabolites contribute to cognition enhancement will be clarified in future studies.

## Data Availability

The data presented in this study are available in the article or in the Supplementary Materials.

## Supplementary Materials

The following are available online at https://www.mdpi.com/article/10.3390/cells11030566/s1, Table S1: Primers for qPCR; Table S2: Gene selection criteria; Table S3: Gene Ontology results for significantly differentially expressed genes stratified by experiment type and tissue.

## Abbreviations

Adgre1 (F4/80)	Adhesion G protein-coupled receptor E1
ANOVA	Analysis of variance
BUNS	Barnes-maze unbiased strategy
Ccl2	C-C motif chemokine ligand 2
Ccl3	C-C motif chemokine ligand 3
Cd68	Cluster of differentiation 68
Cdh1	Cadherin 1
Cldn1	Claudin 1
ENS	Enteric nervous system
ETT	Estimated training time
Gapdh	Glyceraldehyde-3-phosphate dehydrogenase
Gfap	Glial fibrillary acidic protein
HC	Hippocampus
Hmbs	Hydroxymethylbilane synthase
Iba1	Ionized calcium-binding adaptor molecule 1
Ifng	Interferon-gamma
Il10	Interleukin 10
Il1a	Interleukin 1α
Il1b	Interleukin 1β
Il1ra	Interleukin 1 receptor antagonist
Il6	Interleukin 6
Itgam	Integrin alpha M
Muc2	Mucin 2
Ocln	Occludin
PPA	Protein-protein association
P2rx7	Purinergic receptor P2X
qPCR	Quantitative polymerase chain reaction
S100b	S100 calcium-binding protein B
SEM	Standard error of the mean
SI	Social isolation
Tjp1	Tight junction protein 1
Tnf	Tumor necrosis factor-α
VWR	Voluntary wheel running
