# Supplementary material for: Voluntary Wheel Running in Old C57BL/6 Mice Reduces Age-Related Inflammation in the Colon but Not in the Brain
Source: Cells. 2022 Feb 6;11(3):566. doi: 10.3390/cells11030566 (PMC8834481; doi:10.3390/cells11030566)
Supplement: Supplementary file 1 [file cells-11-00566-s001.zip › cells-1429880-supplementary.pdf]

Table S1: Primers for qPCR

Gene	Primer sequence listed fw/rev (5`-3`) or published *[PMID for PubMed]*	Genbank Accesssion Number	Product length [bp]	Start position	
				Fw Primer	Rev Primer
Adgre1 (*F4/80*)	*24796813*	NM_010130.4	164	905	1068
Ccl2	*23301011*	NM_011333.3	90	257	346
Ccl3	*23301011*	NM_011337.2	122	364	485
Cd68	*23301011*	NM_001291058.1	116	847	962
Cdh1	ATCGCCACAGATGATGGTTC<br>CAGTAAAGGGGGACGTGTTG	NM_009864.3	187	1847	2033
Cldn1	AGGTCTGGCGACATTAGTGG<br>AAGAGGGCCTGGCCAAATTC	NM_016674.4	111	600	710
Gapdh	*22028848*	NM_001289726.1	164	944	1107
Gfap	*24796813*	NM_001131020.1	176	636	811
Hmbs	*33782119*	NM_013551.2	106	1132	1237
Iba1 (Aif)	*23301011*	NM_001361501.1	146	478	623
Ifng	CAGCAACAACATAAGCGTCATT<br>GTTGACCTCAAACTTGGCAATAC	NM_008337.4	106	370	475
Il10	*22028848*	NM_010548.2	98	990	1087
Il1ra	CTTCTTCCTCATTTCTGGGTA<br>TGGGTTGTTTTTACTTGGTG	NM_031167	98	2021	2118
Il1a	*22028848*	NM_010554.4	158	879	1036
Il1b (Colon)	ATGCCACCTTTTGACAGTGATG<br>ATGTGCTGCTGCGAGATTTG	NM_008361.4	137	118	254
Il1b (Hippocampus)	*22028848*	NM_008361.4	96	382	477
Il6	GGGAAATCGTGGAAATGAGA<br>TCCAGTTTGGTAGCATCCATC	NM_031168.2	131	257	387
Itgam (Cd11b)	AACTTCACGGCTTCAGAGATG<br>AGGCACTTGAGAGGTTCTGG	NM_001082960	175	2927	3101
Muc2	TGATTGTGTTTCAGGCTCCA<br>GACAGCAGAGCAAGGGACTC	NM_023566.4	94	13358	13451
Ocln	TCGGTACAGCAGCAATGGTA<br>GATATTCCCTGACCCAGTCCT	NM_008756.2	164	1274	1437
P2rx7	AAGAAGTTCCAAGACCCCAGAT<br>TTTCCTCAGATTGTCCAGGAGT	NM_011027.4	96	1427	1522
S100b	CTTCCTTGCTCCCTCTGCT<br>CGACTCTCAGCCTCACCAA	NM_009115.3	171	1199	1369
Tjp1	AAAGCTGTCCCTGTGAGTCC<br>GAATGGCTCCTTGTGGGATA	NM_009386.2	157	5233	5389
Tnf	*22028848*	NM_013693.3	102	313	414

sorted alphabetically

Table S2: Gene selection criteria

	Gene	Gene selection criteria [*PMID for PubMed*]	Database link
Cytokines & Chemokines	*Tnf*	Upregulation in hippocampus of 24 vs. 5 months old male C57BL/6 mice [*24796813*]	http://www.informatics.jax.org/marker/MGI:104798
	Ifng	Upregulation in whole brain of 24-26 vs. 2 months old female BALB/c mice [*10805375*]	http://www.informatics.jax.org/marker/MGI:107656
	Il1a	Upregulation in hippocampus of 24 vs. 5 months old male C57BL/6 mice [*24796813*]	http://www.informatics.jax.org/marker/MGI:96542
	Il1b	Upregulation in hippocampus of 24 vs. 5 months old male C57BL/6 mice [*24796813*]	http://www.informatics.jax.org/marker/MGI:96543
	Il6	Upregulation in whole brain of 24 vs. 6 months old male BALB/c mice [*11431008*]	http://www.informatics.jax.org/marker/MGI:96559
	Il10	Downregulation in hippocampus of 24 vs 3 months old male rats [*15890435*]	http://www.informatics.jax.org/marker/MGI:96537
	Il1ra (Il1rn)	Antagonist of Il1 [*19258032*]	http://www.informatics.jax.org/marker/MGI:96547
	Ccl2	Higher serum Ccl2 protein in 26 vs. 2 months old female C57BL/6 mice [*27069796*]	http://www.informatics.jax.org/marker/MGI:98259
	Ccl3	Upregulation in hippocampus of 24 vs. 5 months old male C57BL/6 mice [*24796813*]	http://www.informatics.jax.org/marker/MGI:98260
Astrocytes	*Gfap*	Upregulation in hippocampus of 24 vs. 5 months old male C57BL/6 mice [*24796813*], mainly expressed in astrocytes and enteric glia cells [*25975510*]	http://www.informatics.jax.org/marker/MGI:95697
	S100b	Upregulation in cerebral cortex in 16 vs. 5 months old male albino mice [*20099023*], expressed in astrocytes (less specific) [*17199889*]	http://www.informatics.jax.org/marker/MGI:98217
Microglia	*Iba1 (Aif1)*	Upregulation in hippocampus of 24 vs. 5 months old male C57BL/6 mice [*24796813*], expressed in microglia [*32848611*]	http://www.informatics.jax.org/marker/MGI:1343098
	Adgre1 (Emr1, *F4/80*)	Upregulation in hippocampus of 24 vs. 5 months old male C57BL/6 mice [*24796813*], expressed in microglia [*32848611*]	http://www.informatics.jax.org/marker/MGI:106912
	Cd68	Upregulation in hippocampus of 24 vs. 5 months old male C57BL/6 mice [*24796813*], expressed in microglia [*32848611*]	http://www.informatics.jax.org/marker/MGI:88342
	Itgam (Cd11b)	Protein upregulated in hippocampus of 21 vs. 4 months old female C57BL/6 mice [*22155499*], expressed in microglia [*32848611*]	http://www.informatics.jax.org/marker/MGI:96607
	P2rx7	Modulates neuroinflammatory processes and has role in CNS diseases as reviewed here [*32848594*], expressed in diverse tissues and cells, such as microglia,	http://www.informatics.jax.org/marker/MGI:1339957

		oligodendrocytes, Schwann cells, and possibly in astrocytes and neurons [*32848594*]; highly expressed in microglia [*28716092*]	
Intestinal barrier function	*Ocln*	Involved in gut barrier function [*19855405*], downregulation in ileum of 24 vs 12 months old male rats [*24243034*]	http://www.informatics.jax.org/marker/MGI:106183
	Cdh1	Involved in gut barrier function [*19855405*]	http://www.informatics.jax.org/marker/MGI:88354
	Tjp1 (ZO-1)	Involved in gut barrier function [*19855405*], downregulation in ileum of 24 vs 12 months old male rats [*24243034*]	http://www.informatics.jax.org/marker/MGI:98759
	Cldn1	Involved in gut barrier function [*19855405*]	http://www.informatics.jax.org/marker/MGI:1276109
	Muc2	Involved in gut barrier function [*19855405*], reduced colonic mucus thickness in 19 vs. 2 months old male C57BL/6 mice [*30723224*]	http://www.informatics.jax.org/marker/MGI:1339364

Table S3: Gene Ontology results for significantly differentially expressed genes stratified by experiment type and tissue.

Only significant results according to uncorrected p-value were reported for colon while the top 5 hits were reported for HC. Query ratio indicates the number of genes found for a GO-term in relation to the size of the input gene list. Background ratio gives the number of all tested genes which belong to a certain GO biological process in relation to all tested genes. The size of the background gene sets was 16 for HC and 21 for colon.

Tissue	Experiment	Change	Gene Ontology ID	Involved Genes	Description	Query Ratio	Background Ratio	P-value	P-adjusted
Hippocampus	Aging	increased (old)	GO:0006911	*Iba1, Itgam*	phagocytosis, engulfment	2/7	2/16	0.175	0.99
			GO:0010575	*Il1a, Il6*	positive regulation of vascular endothelial growth factor production	2/7	3/16	0.400	0.99
			GO:0048661	*Il6, Iba1*	positive regulation of smooth muscle cell proliferation	2/7	3/16	0.400	0.99
			GO:0001781	*Il6, Itgam*	neutrophil apoptotic process	2/7	3/16	0.400	0.99
			GO:0008284	*Il6, S100b, Iba1*	positive regulation of cell population proliferation	3/7	6/16	0.549	0.99
Colon	Aging	increased (old)	GO:0043410	*Tnf, Il6, P2rx7*	positive regulation of MAPK cascade	3/4	3/21	0.003	0.22
			GO:0051384	*Tnf, Il6, Il1ra*	response to glucocorticoid	3/4	4/21	0.012	0.22
			GO:0006954	*Tnf, Il6, Il1ra, P2rx7*	inflammatory response	4/4	8/21	0.012	0.22
			GO:0032731	*Tnf, Il6, P2rx7*	positive regulation of interleukin-1 beta production	3/4	5/21	0.028	0.22
			GO:2000660	*Il6, Il1ra*	negative regulation of interleukin-1-mediated signaling pathway	2/4	2/21	0.029	0.22
			GO:0045837	*Il6, Il1ra*	negative regulation of membrane potential	2/4	2/21	0.029	0.22
			GO:0006953	*Il6, Il1ra*	acute-phase response	2/4	2/21	0.029	0.22
			GO:0045779	*Il6, P2rx7*	negative regulation of bone resorption	2/4	2/21	0.029	0.22
			GO:0060664	*Tnf, Il6*	epithelial cell proliferation involved in salivary gland morphogenesis	2/4	2/21	0.029	0.22
			GO:1904996	*Tnf, Il6*	positive regulation of leukocyte adhesion to vascular endothelial cell	2/4	2/21	0.029	0.22
			GO:1900017	*Tnf, Il6*	positive regulation of cytokine production involved in inflammatory response	2/4	2/21	0.029	0.22
			GO:0043154	*Tnf, Il6*	negative regulation of cysteine-type endopep-tidase activity involved in apoptotic process	2/4	2/21	0.029	0.22
			GO:0033138	*Tnf, Il6*	positive regulation of peptidyl-serine phosphorylation	2/4	2/21	0.029	0.22
			GO:0051897	*Tnf, Il6*	positive regulation of protein kinase B signaling	2/4	2/21	0.029	0.22
			GO:0042127	*Tnf, Il6*	regulation of cell population proliferation	2/4	2/21	0.029	0.22
			GO:0010033	*Tnf, P2rx7*	response to organic substance	2/4	2/21	0.029	0.22
Colon	VWR	decreased (runners)	GO:0090026	*Ccl2, Iba1*	positive regulation of monocyte chemotaxis	2/5	2/21	0.048	0.56
			GO:0006874	*Ccl2, S100b*	cellular calcium ion homeostasis	2/5	2/21	0.048	0.56
			GO:0048662	*Ifng, Iba1*	negative regulation of smooth muscle cell proliferation	2/5	2/21	0.048	0.56
			GO:1904783	*Ifng, Ccl2*	positive regulation of NMDA glutamate receptor activity	2/5	2/21	0.048	0.56
			GO:0051770	*Ifng, Ccl2*	positive regulation of nitric-oxide synthase biosynthetic process	2/5	2/21	0.048	0.56
			GO:2000273	*Ifng, Il10*	positive regulation of signaling receptor activity	2/5	2/21	0.048	0.56
			GO:0045348	*Ifng, Il10*	positive regulation of MHC class II biosynthetic process	2/5	2/21	0.048	0.56
			GO:0001938	*Il10, Ccl2*	positive regulation of endothelial cell proliferation	2/5	2/21	0.048	0.56
			GO:0043524	*Il10, Ccl2*	negative regulation of neuron apoptotic process	2/5	2/21	0.048	0.56
